# Validation and Psychometric Evaluation of the Italian Version of the Fear of COVID-19 Scale

**DOI:** 10.1007/s11469-020-00277-1

**Published:** 2020-05-04

**Authors:** Paolo Soraci, Ambra Ferrari, Francesco A. Abbiati, Elena Del Fante, Rosanna De Pace, Antonino Urso, Mark D. Griffiths

**Affiliations:** 1 Group Cognitive Behavioral Psychology Association, Rome, Italy; 2 Department of Human Science for Education ‘Riccardo Massa’, Università degli Studi di Milano Bicocca, Milan, Italy; 3 Department of Psychology, Università degli Studi di Torino – UNITO, Turin, Italy; 4 ASP - CSM SUD, Reggio Calabria, Italy; 5 Facoltà di Scienze Sociali, Pontificia Università San Tommaso, Rome, Italy; 6 International Gaming Research Unit, Psychology Department, Nottingham Trent University, 50 Shakespeare Street, Nottingham, NG1 4FQ UK

**Keywords:** COVID-19, Disease fear, Italian, Psychometrics, Fear of COVID-19 Scale

## Abstract

**Background:**

The advent of COVID-19 worldwide has led to consequences for people’s health, both physical and psychological, such as fear and anxiety. This is the case in Italy, one of the countries most affected by the pandemic. Given the heightened fear concerning COVID-19 in Italy., the present study analyzed the psychometric properties of the Italian version of the Fear of COVID-19 Scale (FCV-19S).

**Methods:**

The sample comprised 250 Italian participants who were administered Italian versions of the FCV-19S, the Hospital Anxiety and Depression Scale (HADS), and the Severity Measure for Specific Phobia–Adult (SMSP-A). Several psychometric tests were performed to investigate the validity and reliability of the test including confirmatory factor analysis.

**Results:**

Analysis of the data showed satisfactory psychometric characteristics and confirmed the scale’s unidimensional properties. The seven FCV-19S items had acceptable correlations with the test total (from .443 to .784). Furthermore, the loadings on the factor were significant and strong (from .684 to .897). The internal consistency was very good (α = .871). Construct validity for the FCV-19S was supported by significant and positive correlations with the HADS (r=.649) and SMSP-A (r=.703).

**Conclusions:**

The Italian version of the Fear of COVID-19 Scale is valid and reliable in assessing fear of COVID-19 among the general Italian population.

During the last few months of 2019 and the first quarter of 2020, one respiratory infectious disease has unexpectedly become a worldwide emergency, to the point that it was declared a global pandemic by the World Health Organization on March 11, 2020. Novel coronavirus disease 2019 (COVID-19) has affected individuals in 180 countries and territories as of April 2, 2020 (WHO 2020) and has resulted in months-long lockdowns of educational and non-essential business activities in many countries, including Italy.

COVID-19’s symptoms include fever, tiredness, dry cough, myalgia, and dyspnea (Wang et al. 2020), and its mortality rate appears to have fluctuated over the course of the past few months. After initial mortality rates of approximately 15% being reported (Huang et al. 2020), the mortality rate was later found to be between 4.3 and 11% (Wang et al. 2020; Chen et al. 2020). Among the affected countries, Italy currently has the highest mortality rate (7.94%, over 13,000 deaths as of April 2, 2020), followed by Spain (4.50%, over 9000 deaths) and China (3.98%, over 4000 deaths) (Johns Hopkins Center for Systems Science and Engineering 2020).

Together with the disease’s characteristics, such as its being airborne (i.e., perceived as imminent and invisible), further aspects such as uncertainty over patient outcomes, familiarity with infected people, and mandatory change of habits imposed by the governments to protect the population’s health have led many individuals across the globe to experience a generalized sense of fear (Guan et al. 2020; Huang et al. 2020) because similar events are unprecedented in the lifespans of many citizens worldwide. Medical research globally has rightfully focused on the development of an effective vaccine (Dong et al. 2020; Wang et al. 2020), while governments have dedicated themselves to the implementation of strategies for infection control to minimize the spread of the virus.

Nonetheless, a joint effort by medical and psychological healthcare professionals might lead to a better outcome for the entire population affected. In fact, literature regarding past virus outbreaks has already underlined the role of fear and its negative psychosocial consequences in exacerbating the harm of an infectious disease (e.g., Pappas et al. 2009). Often fueled by sensationalistic headlines in the mass media, fear can bring people to oscillate between denial and phobia, while also stigmatizing citizens racially perceived as being the source of the disease (Pappas et al. 2009; Falagas and Kiriaze 2006).

In turn, other psychological disorders such as anxiety and depression have been found associated with fear in previous epidemics, further affecting people’s quality of life negatively (e.g., Ford et al. 2018; Huang et al. 2020). Such consequences appear to be particularly relevant in the context of the present pandemic because social isolation (in this case, resulting from mandatory social distancing policies issued by governments) has been previously shown to be strongly intertwined with anxiety and depression symptoms in both younger and older populations (e.g., Matthews et al. 2019; Santini et al. 2020).

As the fear of coming into contact with individuals who may have been infected has been reported in the context of COVID-19 (Centers for Disease Control and Prevention 2020a, b; Lin 2020), a new psychometric assessment tool assessing an individual’s fear of COVID-19 was recently developed, i.e., the Fear of COVID-19 Scale (FCV-19S), a short and valid robust assessment scale (Ahorsu et al. 2020). Given the degree to which Italy has been hit by the spread of COVID-19, the present study tested the scale among individuals in the Italian population. The aims of the study were to (i) examine the psychometric properties of the Italian the Fear of COVID-19 Scale (FCV-19S) utilizing confirmatory factor analysis (CFA); (ii) assess Fear of COVID-19 in an Italian sample using the Italian FCV-19S; and (iii) confirm whether the Italian FCV-19S is unidimensional as was found in the original validation study by Ahorsu et al. (2020).

## Methods

### Participants and Procedure

A total of 249 participants (age 18 to 76 years) volunteered to take part in the study via an online survey posted in Italian online forums and social network communities (e.g., *Facebook*). The online survey took around 10–15 min to complete. Data collection occurred from 18 March to 21 March 2020. Inclusion criteria for volunteers were being (i) at least 18 years old and (ii) Italian-speaking citizens. All the participants completed the survey anonymously and gave their informed online consent. All procedures conducted were approved by the ethics committee of the Group Cognitive-Behavioral Psychotherapy Association.

### Measures

#### Socio-demographics Parameters

Questions concerning socio-demographic aspects of the participants (e.g., age, gender, educational level) were included in the online survey.

#### Hospital Anxiety and Depression Scale

In order to assess the anxiety and depression levels of participants, the Italian version of the Hospital Anxiety and Depression Scale (HADS) (Costantini et al. 1999) was used. The HADS (Zigmond and Snaith 1983) is a 14-item scale comprising seven items relating to anxiety and seven items relating to depression. Items are answered on a 4-point response format with a total score ranging from 0 to 21 for each of the two subscales. Example items include “I feel as if I am slowed down” (depression) and “I get a sort of frightened feeling like ‘butterflies’ in the stomach” (anxiety). The higher the score, the more severe the anxiety or depression. Cronbach alphas in the present study were very good for the total scale (0.835) and good for the anxiety (0.722) and depression (0.721) subscales.

#### Severity Measure for Specific Phobia—Adult

The Severity Measure for Specific Phobia—Adult (SMSP-A) (Knappe et al. 2013) is a 10-item scale that assesses the severity of specific phobias in individuals aged 18 and older (Italian version: Fossati et al. 2015). Each item asks individuals to rate the severity of their specific phobia during the past 7 days (e.g., “During the past seven days I felt moments of sudden terror, fear, or fright in these situations”). Each item is rated on a 5-point scale from 0 (Never) to 4 (All of the time). The total score can range from 0 to 40 with higher scores indicating greater severity of the specific phobia. Cronbach’s alpha in the present study was very good (.863).

#### Fear of COVID-19 Scale

The FCV-19S (Ahorsu et al. 2020) is a seven-item scale that assesses the fear of COVID-19. The seven items (e.g., “I am most afraid of coronavirus-19”) are rated on a 5-point scale from 1 (strongly disagree) to 5 (strongly agree) with scores ranging from 7 to 35. The higher the score, the greater the fear of COVID-19. For the Italian FCV-19S, the items were independently translated by a mother-tongue translator and internationally accepted practices for translation were employed (Beaton et al. 2000). Additionally, the Italian FCV-19S was piloted on 15 participants of different ages and education levels to investigate if there were any problems in understanding the items themselves (see Appendix). To avoid the effect of the order and the sequence, the order of presentation of scales and the items within the surveys was randomized.

### Statistical Analyses

Univariate normality of the data was verified using the guidelines proposed by Muthén and Kaplan (1985) before the analysis of the sample’s results (i.e., to check if acceptable values for asymmetry/asymmetry and kurtosis were in the range from − 1 to + 1 in the case of normal univariate data distribution). The statistical analyses carried out were as follows: (i) descriptive statistics of the FCV-19S items (i.e., means and standard deviations of the main items); (ii) construct and criterion validity of the Italian FCV-19S; (iii) the reliability of the scale, examined via composite reliability (CR) (e.g., CR values greater than 0.7 are associated with good test reliability; Fornell and Larcker 1981; Netemeyer et al. 2003). The internal consistency of the overall score was calculated using the Cronbach alpha coefficient. In addition, the composite reliability was also taken into consideration, to validate the goodness of the test.

The model fit was examined with the following: the goodness of fit index (GFI), chi-square test (*χ*^2^), degrees of freedom (df), root mean square error of approximation (RMSEA), Confirmatory Fit Index (CFI), standardized root mean square residuals (SRMR), and Tucker-Lewis Index (TLI) fit indices. A good model should have the following characteristics: GFI > 0.90, CFI and TLI > 0.95, RMSEA < 0.06, and SRMR < 0.08 (i.e., Browne and Cudeck 1993) The analysis was carried out using the following statistical packages: FACTOR v. 10.10.01 (Ferrando and Lorenzo-Seva 2017), SPSS Statistics v.25 (IBM Corporation 2017), and “R” software (R Core Team 2014) with the *lavaan* package (Yves Rosseel 2011) and Mplus v.8 (Muthén and Muthén 2017).

## Results

### Descriptive Statistics

The sample (249 participants) comprised 92% female participants (*n* = 229) and 8% male participants (*n* = 20), with a mean age of 34.50 years (SD = 12.21). In terms of education level, 58.7% had a university-level degree, 39% had a high-school degree, and 2.4% had a lower-level educational degree.

### Psychometric Analysis of the Italian FCV-19S

**Fig. 1:**
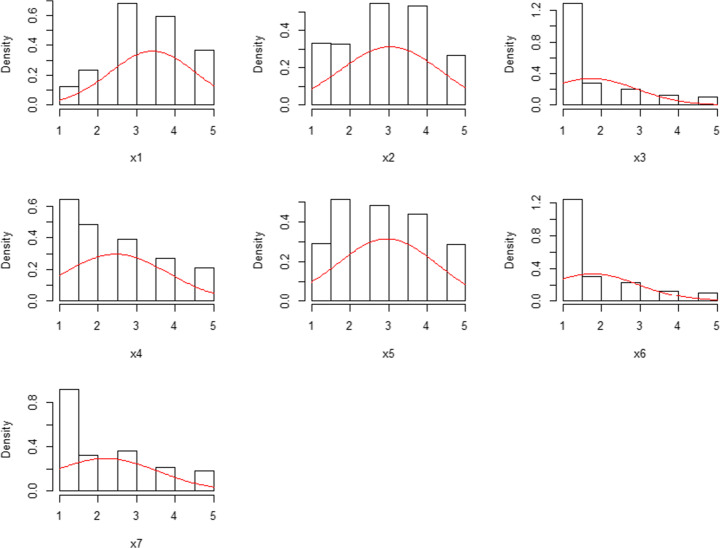
Histograms of the seven FCV-19S items (note: × 1 is Item 1; × 7 is Item 7; the red line refers to the normal distribution curve)

The present study analyzed the distribution of the seven FCV-19S items. Most items (see Fig. 1) were distributed asymmetrically, with the highest frequencies in the lowest values. As for asymmetry and kurtosis, most of the items were distributed in a non-normal way (the items do not fall within the range of ± 1, see Muthén and Kaplan 1985; Tabachnick and Fidell 2007). More specifically, using the Shapiro-Wilk normality test, all items were distributed in a non-normal way (*p* < .01). Moreover, the Italian FCV-19S appeared to have a unidimensional structure (i.e., a single factor). Additionally, by analyzing the Italian FCV-19S, it had eigenvalues > 1 in a single factor model (see Gorsuch 1983) which suggests one factor as the optimal usable model (more specifically, the eigenvalues = 4.60 with a proportion of variance of 0.657).

To investigate and analyze the factorial structure, since there is no unequivocal consensus in the literature (see Bollen and Long 1993; Boomsma 2000), different goodness of fit (GOF) adaptation indices were used to confirm the dimensionality of the FCV-19S. In this specific case, since the items (see Table 1) were distributed in a non-normal way (some items out of the range of ± 1 [see Muthén and Kaplan 1985; Tabachnick and Fidell 2007]), parallel analysis/diagonally weighted least squares method (DWLS, polychoric correlation) was used (Mindrila 2010). The results showed the following: *χ*^2^ = 26.07 (df = 12, *n* = 249), *p* = .011 with *χ*^2^/df = 2.16, the ratio of *χ*^2^ to degrees of freedom [df] < 3 to consider the data-model fit as acceptable (Kline 2011); chi-square test is very sensitive to the size of the sample, so several indices were used (Kline 2016), TLI = 99, CFI = 99, RMSEA = 0.069 (90% confidence interval, lower limit 0.032, upper limit 0.105,* p* = .173, i.e., not significant for *p* = .01) and SRMR = 0.047 (Hu and Bentler 1999). The explained common variance (ECV) was 74.31% (Fornell and Larcker 1981). The factorial validity of FCV-19S was supported by the results (Cerny and Kaiser 1977; Kaiser 1974). The obtained indices were sufficient and all factor loadings were high on all items (min = 0.688, max = 0.897; i.e., λij ≥ 0.50, [Ferguson and Cox 1993]). Furthermore, the modification indices suggest that there was covariance between Item 1 (“I am most afraid of coronavirus-19”) and Item 5 (“When watching news and stories about coronavirus-19 on social media, I become nervous or anxious”) and between Item 2 (“It makes me uncomfortable to think about coronavirus-19”) and Item 7 (“My heart races or palpitates when I think about getting coronavirus-19”).

**Table 1:** Descriptive analysis of the items of the Italian test FCV-19S

Item	Mean	Confidence interval	Variance	Skewness	Kurtosis
Item 1	3.444	3.27–3.62	1.166	− 0.355	− 0.347
Item 2	2.940	2.73–3.15	1.605	− 0.125	− 1.015
Item 3	1.504	1.35–1.66	0.879	1.993	3.366
Item 4	2.411	2.21–2.62	1.597	0.550	− 0.717
Item 5	2.935	2.73–3.14	1.609	0.110	− 1.035
Item 6	1.560	1.42–1.70	0.779	1.477	1.278
Item 7	2.109	1.90–2.32	1.637	0.874	− 0.386

Moreover, a positive relationship between all items (Table 2) was observed (min = 0.443, max = 0.784, all items are statistically significant for *p* = < .01, polychoric correlations). These results indicate that the FCV-19S presented a good fit to the data. In addition, the total score of the FCV-19S on all participants produced the following statistics: mean = 16.86, 95% CI [16.11–17.61], standard deviation = 6.06, skewness = 0.640, kurtosis = − 0.082.

**Table 2:** Standardized variance/covariance matrix (polychoric correlation)

Item	Item 1	Item 2	Item 3	Item 4	Item 5	Item 6	Item 7
Item 1	1.000						
Item 2	0.492**	1.000					
Item 3	0.517**	0.570**	1.000				
Item 4	0.670**	0.443**	0.567**	1.000			
Item 5	0.519**	0.639**	0.629**	0.565**	1.000		
Item 6	0.457**	0.554**	0.675**	0.557**	0.658**	1.000	
Item 7	0.609**	0.522**	0.684**	0.689**	0.740**	0.784**	1.000

**Statistically significant at *p* < .001

After the confirmatory factor analysis, different types of reliability (i.e., internal consistency) and validity (i.e., construct and convergent validity) were investigated. First, construct and criterion validity were tested (Cronbach and Meehl 1955). The total score of the FCV-19S was correlated with variables that have been formerly associated with fear of COVID-19 (i.e., anxiety and depression; Ahorsu et al. 2020). Concurrent validity was supported by the HADS (anxiety and depression levels among participants) and SMSP-A (phobia among participants) as indicated by the significant positive correlations for both scales (*p* < .001). More specifically, the FCV-19S positively correlated with the HADS (*r* = .649) and the SMSP-A (*r* = .703). To analyze the reliability of the measure and internal consistency, Cronbach’s alpha, Factor Determinacy Index, and composite reliability (Raykov 1997) were used. Cronbach’s alpha in the present study was 0.871 and could not be improved by removing any items. The Factor Determinacy Index was 0.966 and the composite reliability was 0.907. Furthermore, age was negatively correlated with the FCV-19S test but was non-significant (*r* = − .03, *p* = .533).

## Discussion

The present study investigated the psychometric properties of the Italian Fear of COVID-19 Scale (FCV-19S). Results indicated a stable unidimensional structure of the Italian FCV-19S, confirming the findings of the original validation study (Ahorsu et al. 2020). Psychometric analyses showed the Italian FCV-19S’s good internal reliability and consistency. Construct validity was confirmed by the significant correlation with HADS (which assesses the general level of anxiety and depression) and SMSP-A (which assesses the level of severity of the specific phobia). This is in accordance with previous literature (e.g., Brannon and Schuyler 2000; American Psychiatric Association 2013) according to which specific phobias are often comorbid with anxiety (which is unsurprising given that phobias can be considered as a defense by individuals from anxiety; Greenson 1959). In fact, a significant positive correlation between FCV-19S scores and scores on the HADS and SMSP-A was observed, confirming the scale’s convergent validity.

Further observations demonstrated that the fear response pattern was not significantly influenced by the participant’s age. This suggests that the Italian FCV-19S can be used to assess psychological issues caused by COVID-19 diffusion among all ages. Although no formal diagnoses concerning mood disorders were obtained (e.g., anxiety, depression), scores on the FCV-19S were significantly and positively related to scores assessing depression and anxiety (HADS) and the severity of the specific phobia (SMSP-A); therefore, individuals with severe fear of COVID-19 may be affected by these disorders co-morbidly. This is in accordance with previous literature indicating that, during long periods of infectious epidemics, individuals’ psychophysical health can be affected by negative psychological states (e.g., anxiety, depression, and phobias; Duncan et al. 2009; Pappas et al. 2009; Ropeik 2004). Besides generating concerns on a physical and psychological healthcare level, COVID-19 has also resulted in social issues, which have been previously associated with an acceleration of an infectious disease’s spread (Centers for Disease Control and Prevention 2020a, b; Bloom and Cadarette 2019). For instance, an epidemic can overload a nation’s healthcare system, especially in poor public health contexts (Bloom and Cadarette 2019), limiting the ability of operators to deal with the problem and increasing the stress levels of both citizens and healthcare workers. In turn, stress has been previously shown to worsen both the physical and mental health of individuals, often resulting in increased use of alcohol, tobacco, or other drugs (Centers for Disease Control and Prevention 2020c) and negatively affecting the immune system, making people more vulnerable to disease (e.g., Morey et al. 2015). Moreover, fear experienced during an epidemics has been previously associated with the stigmatization of citizens perceived as being the source of the disease, with the risk of resulting in scuffles or, in extreme cases, civil conflicts (Pappas et al. 2009; Falagas and Kiriaze 2006). Therefore, the Italian version of FCV-19S could help the general public to better understanding emotional factors correlated with the pandemic and (with a joint effort by those working in medical and psychological healthcare) ultimately lead to a better health outcome for the entire population affected.

The findings of the present study should be viewed in light of some limitations. First, the participant pool only comprised a self-selected sample from the general Italian population with a majority being female (and therefore, tests for gender differences were not possible). Second, no formal diagnosis of mood disorders was undertaken. Third, it cannot be excluded that social desirability factors might have influenced participant responses to the questionnaire. Further investigation on bigger and more representative samples of Italian participants is needed to confirm the preliminary results provided by the present study (e.g., a nationally representative sample with more male participants). However, total scores on the FCV-19S were comparable across all ages, which suggests that the Italian FCV-19S is a good psychometric instrument to be used in assessing fears of COVID-19 among Italian individuals.

Future studies should also evaluate if individuals with underlying medical conditions associated with a higher risk of death from COVID-19 (e.g., diabetes, hypertension, coronary heart disease, pre-existing respiratory conditions) may experience increased levels of COVID-19 fear. Moreover, future research including a larger and more representative sample should further investigate the apparent covariance between Item 1 and Item 5 and between Item 2 and Item 7. Preliminary covariance results may, in fact, be due to different factors, such as (i) an underlying factor that was not considered in the present model, (ii) the sample size, (iii) the imbalance of the sample’s gender composition, (iv) idiosyncratic characteristics of the sample, and (v) the semantic similarity between the items in Italian context (Whittaker 2012). Nonetheless, the psychometric testing of the Italian FCV-19S demonstrates that the instrument is psychometrically robust and assesses a unidimensional construct. In short, the Italian FCV-19S is a reliable and valid tool for assessing the severity of fear of COVID-19 among Italian adults.

## Appendix. Italian version of the Fear of COVID-19 Scale

1. Ho molta paura del coronavirus-19
2. Mi rende inquieto (ansioso/nervoso) pensare al coronavirus-19
3. Le mie mani iniziano a sudare quando penso al coronavirus-19
4. Ho paura di perdere la vita a causa del coronavirus-19
5. Quando guardo le notizie e le storie sul coronavirus-19 sui social media, divento nervoso o ansioso.
6. Non riesco a dormire perché mi preoccupo di contrarre (o avere) il coronavirus-19
7. Il mio cuore batte forte o palpita quando penso di poter contrarre il coronavirus-19
